# CSM-lig: a web server for assessing and comparing protein–small molecule affinities

**DOI:** 10.1093/nar/gkw390

**Published:** 2016-05-05

**Authors:** Douglas E.V. Pires, David B. Ascher

**Affiliations:** 1 Centro de Pesquisas René Rachou, Fundação Oswaldo Cruz, Belo Horizonte, 30190-002, Brazil; 2 Department of Biochemistry, University of Cambridge, Cambridge, CB2 1GA, UK; 3 Department of Biochemistry, University of Melbourne, Victoria 3010, Australia

## Abstract

Determining the affinity of a ligand for a given protein is a crucial component of drug development and understanding their biological effects. Predicting binding affinities is a challenging and difficult task, and despite being regarded as poorly predictive, scoring functions play an important role in the analysis of molecular docking results. Here, we present CSM-Lig (http://structure.bioc.cam.ac.uk/csm_lig), a web server tailored to predict the binding affinity of a protein-small molecule complex, encompassing both protein and small-molecule complementarity in terms of shape and chemistry via graph-based structural signatures. CSM-Lig was trained and evaluated on different releases of the PDBbind databases, achieving a correlation of up to 0.86 on 10-fold cross validation and 0.80 in blind tests, performing as well as or better than other widely used methods. The web server allows users to rapidly and automatically predict binding affinities of collections of structures and assess the interactions made. We believe CSM-lig would be an invaluable tool for helping assess docking poses, the effects of multiple mutations, including insertions, deletions and alternative splicing events, in protein-small molecule affinity, unraveling important aspects that drive protein–compound recognition.

## INTRODUCTION

Interactions between small molecules and proteins mediate many essential biological effects, and are a vital consideration in the development of new drugs. Molecular docking can be used not only to facilitate the drug development process (1–3), but can provide useful insights into protein function prediction and other important problems (4–6).

The first stage of docking involves the generation of poses to reflect the position, orientation and conformation of a given molecule docked to the target, with current algorithms having good predictive power. These poses are then scored based upon a prediction of how tightly the ligand interacts with and is bound to the target. These scoring functions have been developed around force-fields and energy-based approaches (7–12), knowledge-based (13,14) and empirical approaches (15), however it is widely recognized that the inaccuracies of current scoring functions are a major hurdle to achieving reliability in docking (16–18). This might be, among other factors, due to certain simplifications and assumptions while designing a scoring function, usually guided by the necessity of reducing computational load, which means that certain physical processes and aspects that are relevant for understanding small-molecule binding are not simulated, introducing some of this inaccuracy. Novel approaches for predicting protein–compound affinities in a effective and scalable way are, therefore, of great need.

We have successfully used a class of graph-based signatures, called cutoff scanning matrix (CSM), to represent the three-dimensional (3D) environment of proteins and small ligands (19,20). These have been used successfully as evidence to train accurate predictors of protein function and structural classification (20), receptor-based ligand prediction (21), the effects of mutation on stability and binding to proteins, nucleic acids and ligands (22) and the pharmacokinetic properties of small molecules (23).

We show here that the CSM signatures can be used successfully to accurately predict the binding affinity of small molecules to proteins. By comparing its performance, we show that CSM-Lig performs as well as or better than several other widely used methods and scoring functions, and is efficient, facilitating its use as a part of large scale approaches. We also provide a freely accessible and user-friendly web interface in http://structure.bioc.cam.ac.uk/csm_lig.

## MATERIALS AND METHODS

### Graph-based structural signatures

The CSM algorithm defines a class of graph-based signatures by modeling proteins/small molecule recognition as graphs where atoms are seen as nodes and their interactions as edges, and extracting distance patterns between its components.

Atoms are labeled with eight pharmacophore types as in (22), based on their physicochemical characteristics and a cumulative distribution of distances between atoms (within the protein-binding pocket and between the small molecule and protein) per pharmacophore pair is generated.

Complementary small molecule properties were calculated using the Python RDKit library and are also included in the signature. The complete list of used properties is available in Supplementary Material (Table S1).

**Figure 1.**
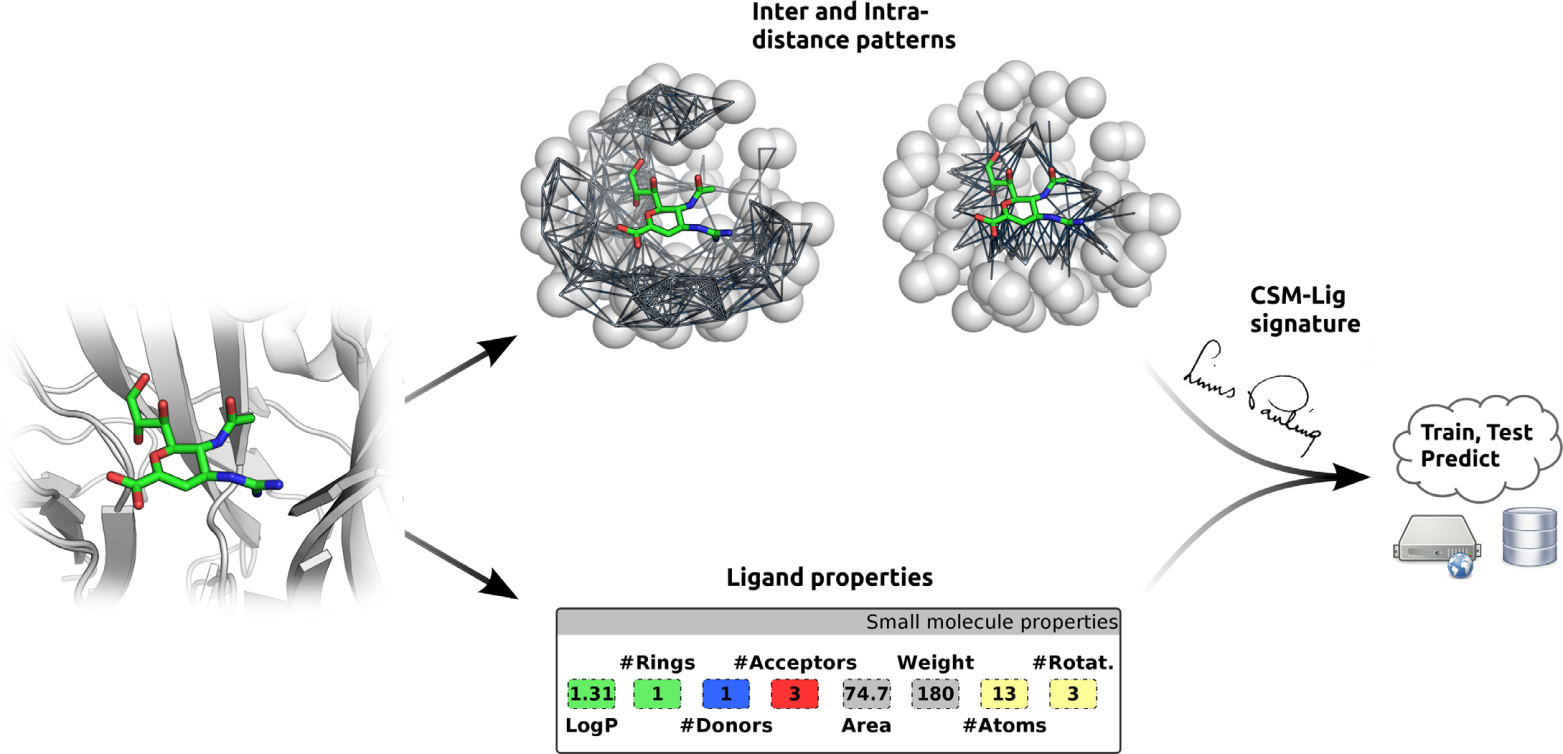
CSM-Lig workflow. Based on a given protein-small molecule structure, CSM-Lig extracts the environment of the binding site from which structural signatures are derived, as well as physicochemical properties of the ligand. This information, together with experimentally measured protein small molecule binding affinities from PDBind are used to train and test predictive models using machine learning.

These two sets of information encode the shape/chemical composition of the receptor, the nature of the protein–ligand interactions as well as the type of the ligand, which are then used as evidence to train and test predictive methods with machine learning, using Gaussian Processes (24), with available experimental data in the literature. Figure 1 shows a workflow of the CSM-Lig methodology.

### Data sets

The PDBbind database (25) is a comprehensive collection of experimentally measured affinities (*K*_i_, *K*_D_ and IC 50) for different types of biomolecular complexes deposited in the Protein Data Bank, including protein-small molecule binding data.

The database provides high-quality data sets specifically designed for evaluating docking methods and scoring functions, called ‘refined’ and ‘core’ sets. These were defined by a set of general rules to include high-resolution structures, for which *K*_i_ or *K*_D_ are available for non-covalently bound ligands. Further details on the criteria for filtering these data sets can be found in (26,27).

The core set is composed by complexes derived from the refined set clustered by sequence similarity (90%), where, for each cluster three complexes are chosen: highest, lowest and middle binding affinity, generating a very diverse, representative and non-redundant set with a wide spread of binding affinities.

The complexes of the refined set that are not present in the core set are used as training set, while the core set is used as a blind test. Figure S1 of Supplementary Material shows the histogram of affinities for each release and its core set (used in this work as blind tests). CSM-Lig was initially built based on the PDBbind 2007 and 2013 releases, which were used for its performance evaluation, and subsequently on the 2014 release. The CSM-Lig predictive model was evaluated in 10-fold cross validation and using the core sets of each release as blind tests. The training/test sets for the PDBbind 2007, 2013 and 2014 releases used in this work are composed by 1301/195, 2860/184 and 3167/184 complexes, respectively.

In order to directly and impartially compare the performance of the CSM-Lig methodology against other well established approaches, the model built upon the 2007 release was used to compare against a broad range of alternative methods that have been built and tested using this release. In order to assess and compare the predictive performance of CSM-Lig with other methods we have used a set of evaluation metrics including Pearson and Spearman correlation coefficients (on the whole data set and across 90% of the data) as well as the Standard Deviation. The significance of the differences in correlations between CSM-Lig and alternative approaches was determined by the Fisher r-to-z transformation. In order to compare the standard deviations of the different methods, the F-test was used. In both cases a significance threshold of *P* ≤ 0.05 was employed.

## WEB SERVER

**Figure 2.**
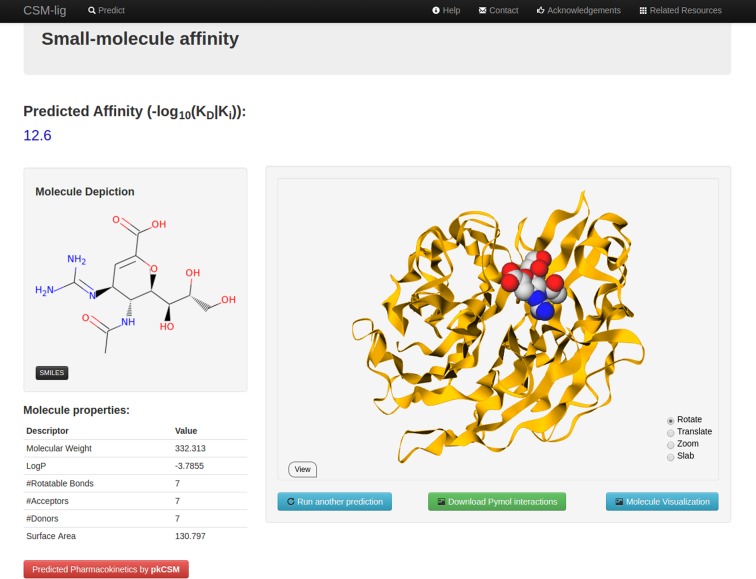
Web server interface. The figure depicts the result page for a single protein-small molecule affinity prediction mode for CSM-Lig, which shows the numerical binding affinity prediction as the −*log*_10_(*K*_D_|*K*_i_), an interactive GLMol session of the ligand binding location, a downloadable pymol session file to explore the interactions made by the ligand and also a link for the predicted pharmacokinetic and toxicity properties of the ligand calculated by pkCSM (23).

We have implemented CSM-Lig via a user-friendly webserver freely available at http://structure.bioc.cam.ac.uk/csm_lig (Figure S2 of Supplementary Material). The server front-end was built using Bootstrap framework version 2.0, while the back-end was built in Python via the Flask framework (version 0.10.1), running on a Linux server. It allows users to upload protein–ligand complexes (in PDB format) or a compressed file of multiple protein–ligand complexes, for which the predicted binding affinities will be calculated. These could include different poses of the same complex, different ligands or multiple different proteins. For multiple complexes, the predicted affinities are shown in an interactive table and can be downloaded as a tab-separated file. In addition, a Pymol session file showing the interactions made by the ligand within the structure (Figure S3 of Supplementary Material) will be generated using Arpeggio (Jubb H and Blundell TL, Unpublished Data), and made available to download. For single predictions a WebGL interactive molecule visualization of the uploaded complex is also shown. Figure 2 depicts the result page for a single complex prediction. No user information is retained on the system after being uploaded by the user. Water molecules present in the uploaded PDB files are removed prior to calculation. As part of the PDBbind curated data set used in this study, all other Hetatoms, including cofactors, are removed from the structures. Since these were not present in the training set for CSM-lig, the server automatically removes them from the files.

## RESULTS

CSM-Lig was first built and evaluated on the widely used 2007 and 2013 releases of PDBbind. For the two releases, CSM-lig achieved a Pearson's correlation coefficients of 0.82 and 0.86, Spearman correlations of 0.83 and 0.87 and standard deviation of 0.94 and 0.87, respectively, on 10-fold cross validation. Over the PDBbind core set, a blind test of 195 diverse complexes with binding affinities ranging from millimolar to picomolar that has been used to benchmark different approaches, the models showed strong Pearson's correlations of 0.75 and 0.80 (Spearman correlations of 0.76 and and 0.81) and with a small spread in errors between the CSM-Lig predictions and experimentally measured affinities (standard deviation of 1.617 and 1.440, respectively). Figure S4 of Supplementary Material shows the regression plots for the 2007 and 2013 releases. We have evaluated whether the accuracy of the method was biased toward certain ligand properties, however, no significant correlation has been identified (Figure S5 of Supplementary Material).

Comparing the performance of CSM-Lig built upon the 2007 PDBbind data set against previously benchmarked approaches that used this data set (28), we observed that CSM-Lig performed as good or better than well established scoring functions and predictors (Table 1). The Pearson and Spearman correlations (0.751 and 0.761, respectively) and the standard deviation (1.617) achieved by CSM-Lig on the 2007 data set were significantly better than all other methods, apart from RF-Score, which performed comparably and was not statistically different.

**Table 1.** Comparative performance between CSM-Lig and similar methods and scoring functions on the PDBbind 2007 core set

Method/Scoring Function	R_*P*_	R_*S*_	SD
CSM-Lig	0.751	0.761	1.617
RF-Score::Elem-v2	0.803	0.797	1.540
RF-Score::Elem-v1	0.776	0.762	1.580
X-Score::HMScore	0.644*	0.705	1.830**
DrugScore^*CSD*^	0.569*	0.627*	1.960**
SYBYK::ChemScore	0.555*	0.585*	1.980**
DS::PLP1	0.545*	0.588*	2.000**
GOLD::ASP	0.534*	0.577*	2.020**
SYBYL::G-Score	0.492*	0.536*	2.080**
DS::LUDI3	0.487*	0.478*	2.090**
DS::LigScore2	0.464*	0.507*	2.120**
GlideScore-XP	0.457*	0.435*	2.140**
DS::PMF	0.445*	0.448*	2.140**
GOLD::ChemScore	0.441*	0.452*	2.150**
NHA	0.431*	0.517*	2.150**
SYBYL::D-Score	0.392*	0.447*	2.190**
IMP::RankScore	0.322*	0.348*	2.250**
DS::Jain	0.316*	0.346*	2.240*
GOLD::GoldScore	0.295*	0.322*	2.290**
SYBYL::PMF-Score	0.268*	0.273*	2.290**
SYBYL::F-Score	0.216*	0.243*	2.350**

Pearson (R_*P*_) and Spearman Correlations (R_*S*_), as well as Standard Deviation (SD) are given. Results from similar methods directly obtained from Balester *et al*. (2014). Full references of the listed methods can be found on Table S2 of Supplementary Material. *P ≤ 0.05 compared to CSM-Lig by Fisher *r* − *to* − $z$ transformation. **P ≤ 0.05 compared to CSM-Lig by F-test.

**Figure 3.**
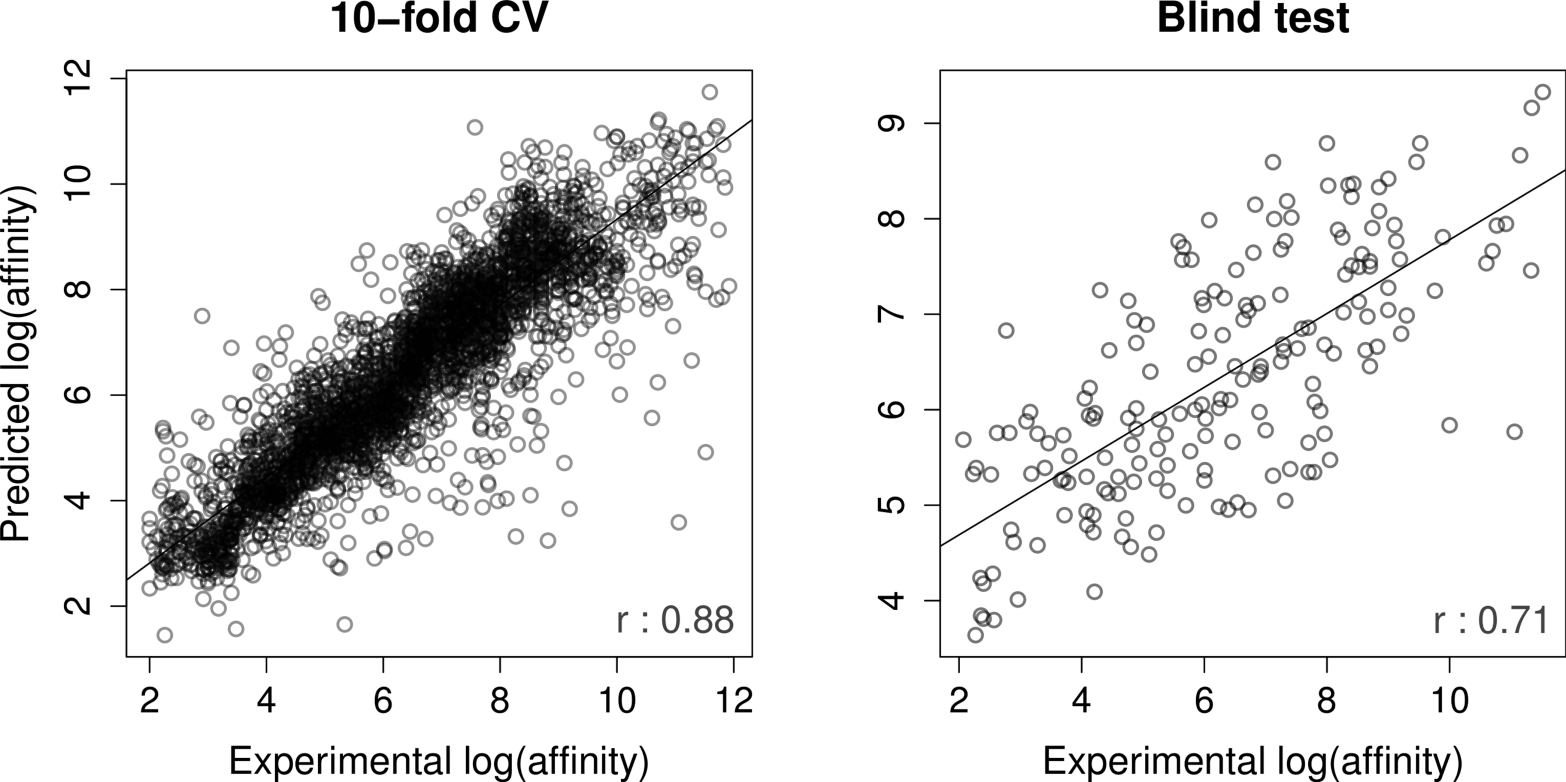
Regression plot between experimental and predicted affinities by CSM-lig on the PDBbind 2014 release. The graph on the left-hand side depicts the performance of CSM-lig over 10-fold cross validation, achieving a Pearson's correlation of 0.88 (using 3167 protein–ligand complexes). The performance in blind test for this release was 0.71 (composed by 184 protein–ligand complexes).

The CSM-Lig server was built and evaluated using the latest release of PDBbind, 2014, and the updated core sets as a blind test. CSM-lig achieved a Pearson's correlation coefficient of 0.88 on 10-fold cross validation and 0.71 for the core set, as a blind test (Spearman correlation of 0.89 and 0.70, respectively). Figure 3 shows a regression plot between experimental and predicted affinities for the PDBbind 2014 release.

## CONCLUSIONS

We present a new approach, CSM-Lig, for predicting the binding affinity of a protein–small molecule complex. CSM-Lig relies on graph-based signatures, was successfully applied and evaluated in different predictive tasks and was shown to outperform earlier methods. The results achieved by CSM-Lig support the idea that the binding affinity of a small molecule can be correlated with the atomic distance patterns surrounding a bound ligand.

## Supplementary Material

SUPPLEMENTARY DATA

## References

[1] Sigurdardottir A., Winter A., Sobkowicz A., Fragai M., Chirgadze D., Ascher D., Blundell T., Gherardi E. (2015). Exploring the chemical space of the lysine-binding pocket of the first kringle domain of hepatocyte growth factor/scatter factor (HGF/SF) yields a new class of inhibitors of HGF/SF-MET binding. Chem. Sci..

[2] Albiston A.L., Morton C.J., Ng H.L., Pham V., Yeatman H.R., Ye S., Fernando R.N., De Bundel D., Ascher D.B., Mendelsohn F.A. (2008). Identification and characterization of a new cognitive enhancer based on inhibition of insulin-regulated aminopeptidase. FASEB J..

[3] Chai S.Y., Yeatman H.R., Parker M.W., Ascher D.B., Thompson P.E., Mulvey H.T., Albiston A.L. (2008). Development of cognitive enhancers based on inhibition of insulin-regulated aminopeptidase. BMC Neurosci..

[4] Hermans S.J., Ascher D.B., Hancock N.C., Holien J.K., Michell B.J., Chai S.Y., Morton C.J., Parker M.W. (2015). Crystal structure of human insulin-regulated aminopeptidase with specificity for cyclic peptides. Prot. Sci..

[5] Ascher D.B., Wielens J., Nero T.L., Doughty L., Morton C.J., Parker M.W. (2014). Potent hepatitis C inhibitors bind directly to NS5A and reduce its affinity for RNA. Sci. Rep..

[6] Ye S., Chai S.Y., Lew R.A., Ascher D.B., Morton C.J., Parker M.W., Albiston A.L. (2008). Identification of modulating residues defining the catalytic cleft of insulin-regulated aminopeptidase. Biochem. Cell Biol..

[7] Vanommeslaeghe K., Hatcher E., Acharya C., Kundu S., Zhong S., Shim J., Darian E., Guvench O., Lopes P., Vorobyov I. (2010). CHARMM general force field: A force field for drug-like molecules compatible with the CHARMM all-atom additive biological force fields. J. Comput. Chem..

[8] Huey R., Morris G.M., Olson A.J., Goodsell D.S. (2007). A semiempirical free energy force field with charge-based desolvation. J. Comput. Chem..

[9] Wang J., Wolf R.M., Caldwell J.W., Kollman P.A., Case D.A. (2004). Development and testing of a general amber force field. J. Comput. Chem..

[10] Jain T., Jayaram B. (2005). An all atom energy based computational protocol for predicting binding affinities of protein–ligand complexes. FEBS Lett..

[11] Jain T., Jayaram B. (2007). Computational protocol for predicting the binding affinities of zinc containing metalloprotein–ligand complexes. Proteins.

[12] Brylinski M. (2013). Nonlinear scoring functions for similarity-based ligand docking and binding affinity prediction. J. Chem. Inf. Model..

[13] Muegge I. (2006). PMF scoring revisited. J. Med. Chem..

[14] Velec H.F., Gohlke H., Klebe G. (2005). DrugScoreCSD knowledge-based scoring function derived from small molecule crystal data with superior recognition rate of near-native ligand poses and better affinity prediction. J. Med. Chem..

[15] Friesner R.A., Banks J.L., Murphy R.B., Halgren T.A., Klicic J.J., Mainz D.T., Repasky M.P., Knoll E.H., Shelley M., Perry J.K. (2004). Glide: a new approach for rapid, accurate docking and scoring. 1. Method and assessment of docking accuracy. J. Med. Chem..

[16] Warren G.L., Andrews C.W., Capelli A.-M., Clarke B., LaLonde J., Lambert M.H., Lindvall M., Nevins N., Semus S.F., Senger S. (2006). A critical assessment of docking programs and scoring functions. J. Med. Chem..

[17] Yuriev E., Holien J., Ramsland P.A. (2015). Improvements, trends, and new ideas in molecular docking: 2012-2013 in review. J. Mol. Recognit..

[18] Grinter S.Z., Zou X. (2014). Challenges, applications, and recent advances of protein-ligand docking in structure-based drug design. Molecules.

[19] da Silveira C.H., Pires D. E.V., Melo-Minardi R.C., Ribeiro C., Veloso C.J.M., Lopes J.C.D., Meira W., Neshich G., Ramos C. H.I., Habesch R. (2009). Protein cutoff scanning: A comparative analysis of cutoff dependent and cutoff free methods for prospecting contacts in proteins. Proteins.

[20] Pires D. E.V., Melo-Minardi R.C., Santos M.A., da Silveira C.H., Santoro M.M., Meira W. (2011). Cutoff Scanning Matrix (CSM): structural classification and function prediction by protein inter-residue distance patterns. BMC Genomics.

[21] Pires D. E.V., de Melo-Minardi R.C., da Silveira C.H., Campos F.F., Meira W. (2013). aCSM: noise-free graph-based signatures to large-scale receptor-based ligand prediction. Bioinformatics.

[22] Pires D. E.V., Ascher D.B., Blundell T.L. (2014). mCSM: predicting the effects of mutations in proteins using graph-based signatures. Bioinformatics.

[23] Pires D. E.V., Blundell T.L., Ascher D.B. (2015). pkCSM: predicting small-molecule pharmacokinetic and toxicity properties using graph-based signatures. J. Med. Chem..

[24] Rasmussen C.E., Williams C.K. (2006). Gaussian processes for machine learning.

[25] Liu Z., Li Y., Han L., Li J., Liu J., Zhao Z., Nie W., Liu Y., Wang R. (2014). PDB-wide collection of binding data: current status of the PDBbind database. Bioinformatics.

[26] Li Y., Liu Z., Li J., Han L., Liu J., Zhao Z., Wang R. (2014). Comparative assessment of scoring functions on an updated benchmark: 1. Compilation of the test set. J. Chem. Inf. Model..

[27] Li Y., Han L., Liu Z., Wang R. (2014). Comparative assessment of scoring functions on an updated benchmark: 2. Evaluation methods and general results. J. Chem. Inf. Model..

[28] Ballester P.J., Schreyer A., Blundell T.L. (2014). Does a more precise chemical description of protein–ligand complexes lead to more accurate prediction of binding affinity?. J. Chem. Inf. Model..

