# Supplementary material for: CSM-lig: a web server for assessing and comparing protein–small molecule affinities
Source: Nucleic Acids Res. 2016 May 5;44(Web Server issue):W557–61. doi: 10.1093/nar/gkw390 (PMC4987933; doi:10.1093/nar/gkw390)
Supplement: SUPPLEMENTARY DATA [file supp_44_W1_W557__index.html]

CSM-lig: a web server for assessing and comparing protein–small molecule affinities — CSM-lig: a web server for assessing and comparing protein–small molecule affinities — SUPPLEMENTARY DATA 

# CSM-lig: a web server for assessing and comparing protein–small molecule affinities

## SUPPLEMENTARY DATA

- SUPPLEMENTARY DATA
