# Supplementary material for: CSM-lig: a web server for assessing and comparing protein–small molecule affinities
Source: Nucleic Acids Res. 2016 May 5;44(Web Server issue):W557–61. doi: 10.1093/nar/gkw390 (PMC4987933; doi:10.1093/nar/gkw390)
Supplement: SUPPLEMENTARY DATA [file supp_gkw390_nar-00524-web-b-2016-File009.pdf]

CSM-lig: a web server for assessing and comparing protein-small molecule affinities

Douglas E. V. Pires*, David B. Ascher*

Supplementary Material

1 Figures

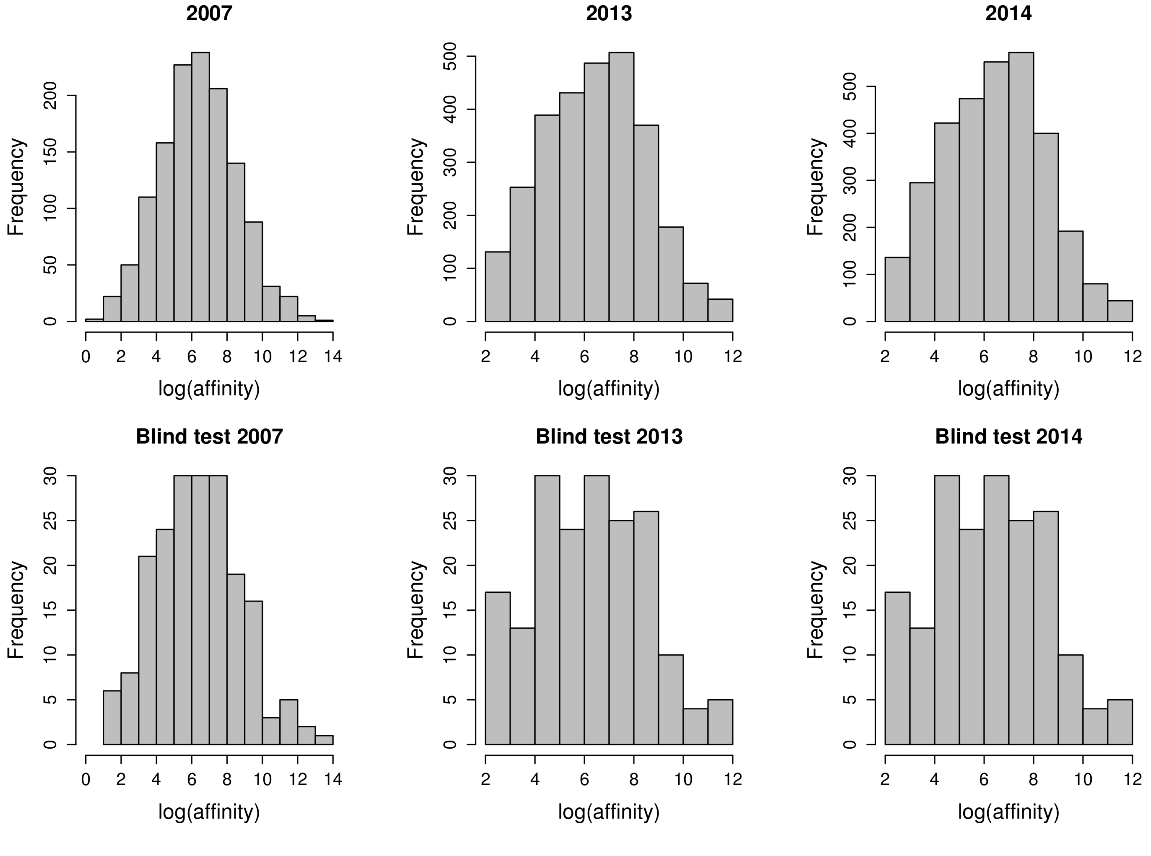

Figure 1: Distribution of experimental affinities for protein-ligand complexes in different PDBbind releases. The distribution of affinities for the blind tests (core sets) are also shown.).

*Email: douglas.pires@cpqrr.fiocruz.br; Correspondence may also be addressed to dascher@svi.edu.au

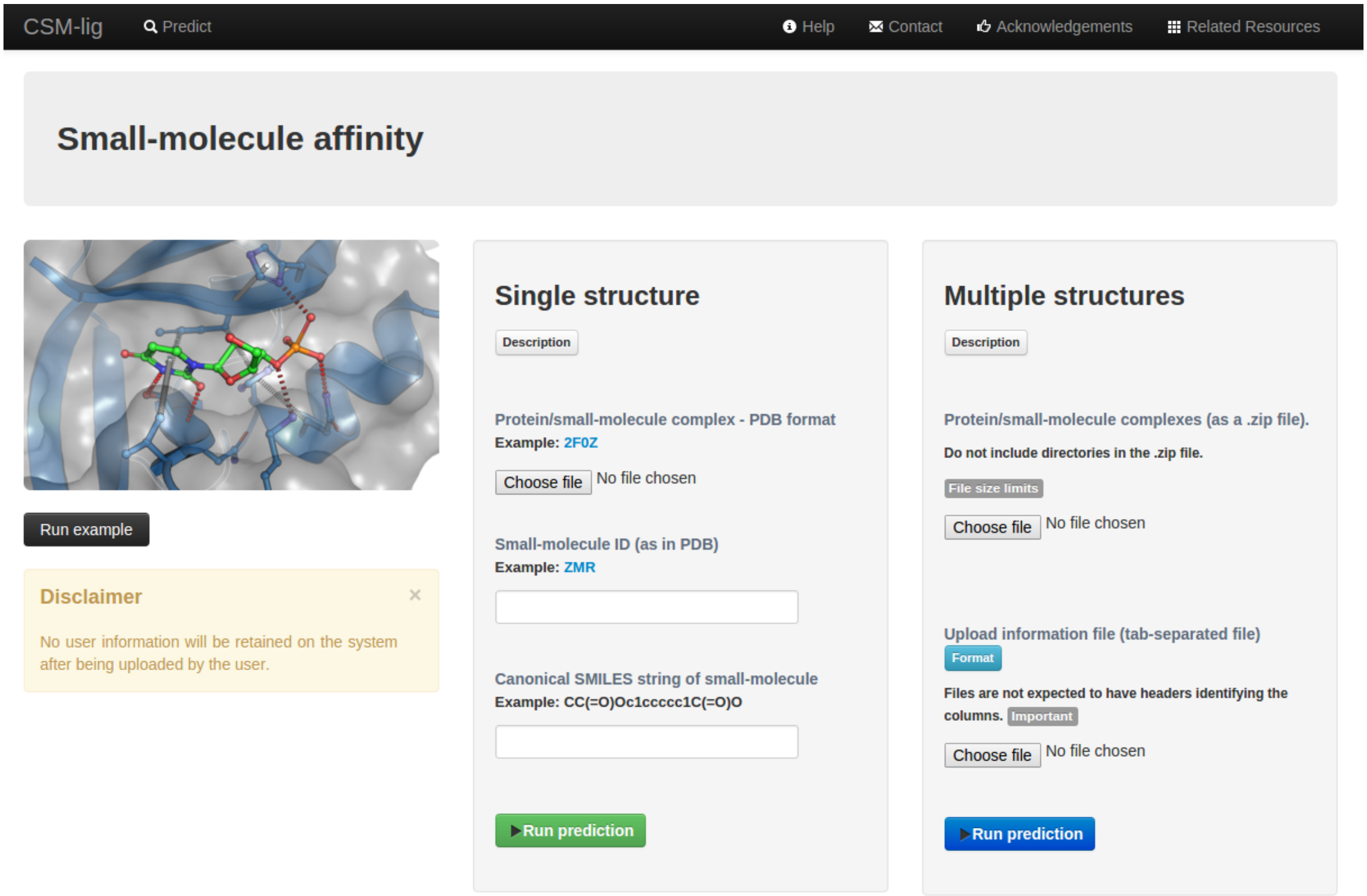

Figure 2: CSM-Lig job submission interface. Users have the option to perform CSM-Lig predictions on a single uploaded structure or in multiple structures uploaded in a compressed file.

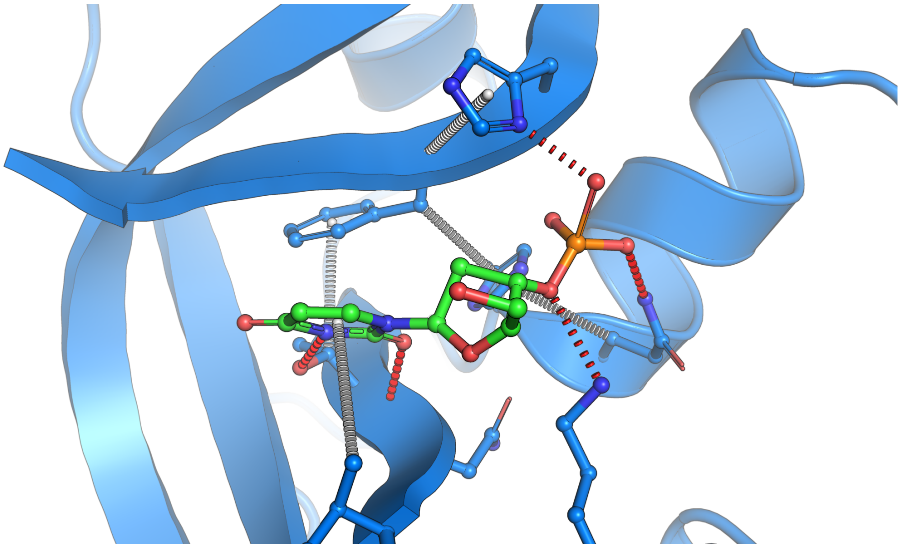

Figure 3: Example of visualization of protein-small molecule interactions generated by Arpeggio (Jubb H and Blundell TL, Unpublished Data) and made available on CSM-Lig. Hydrogen bonds are shown as red dashes and carbon-pi interactions as grey dashes. PDB ID: 1W4P was used.

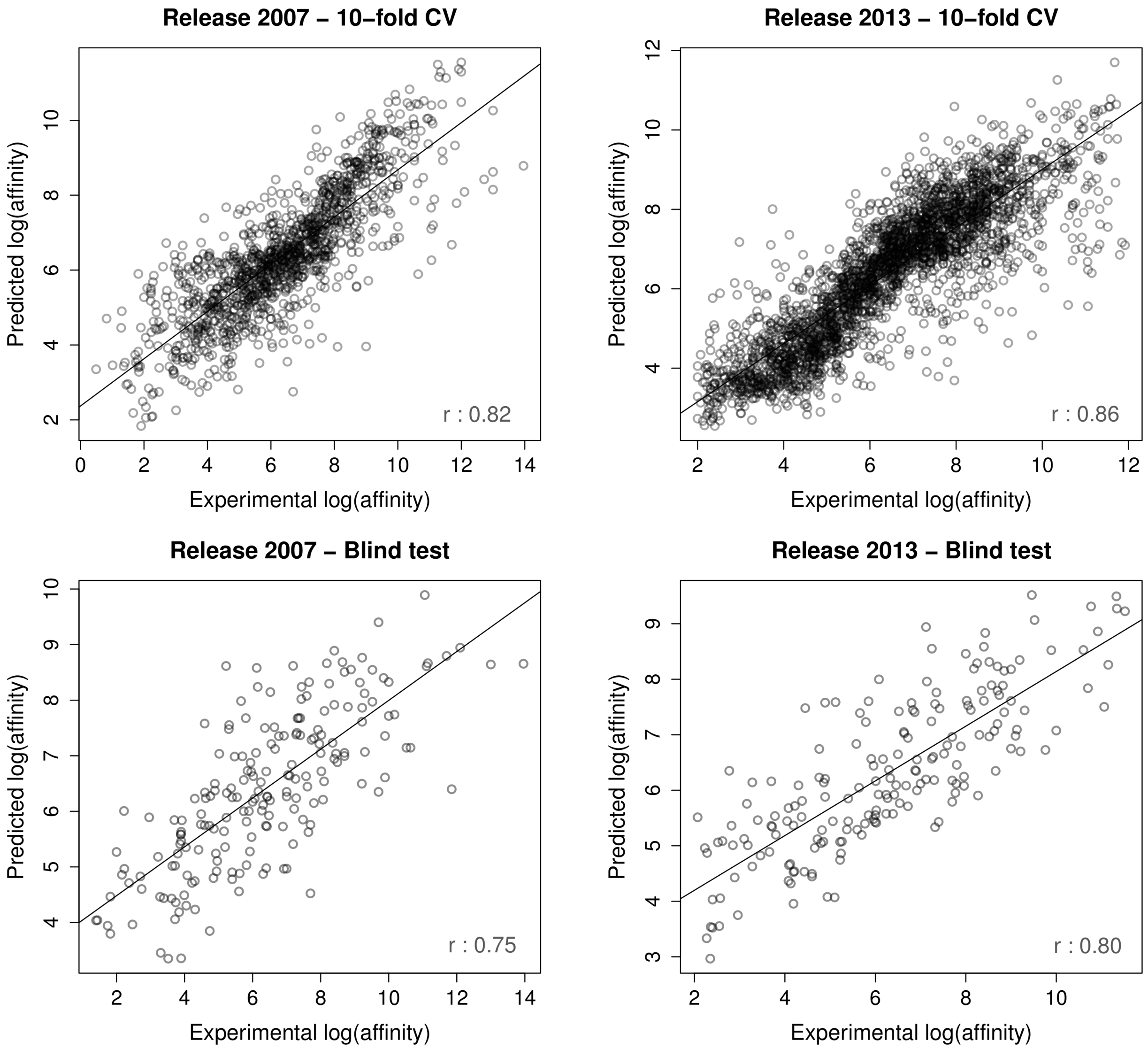

Figure 4: Regression plot between experimental and predicted affinities by CSM-lig on the PDBbind 2007 and 2013 releases. The graph on the top depicts the performance of CSM-lig over 10-fold cross validation, achieving a Pearsons correlation of 0.82 on release 2007 and 0.86 on release 2013. The performance in blind tests for these the 2007 and 2013 releases was 0.75 and 0.80, respectively.

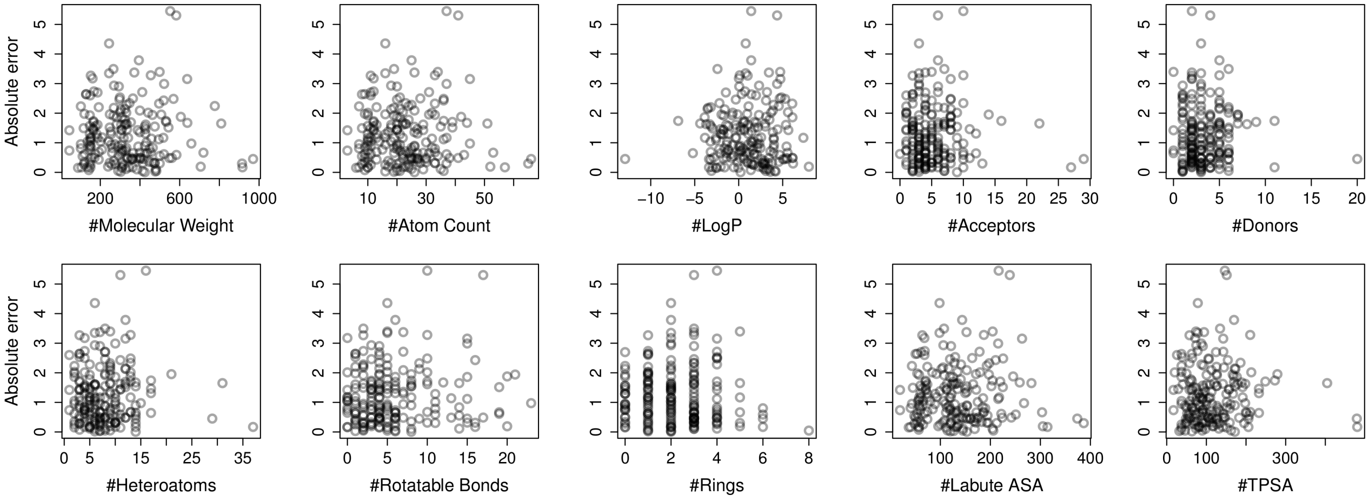

Figure 5: Regression plot between absolute errors of predictions and small-moluce properties on the PDBbind 2007 blind test. No significant correlation has been identified.

2 Tables

Table 1: List of molecular properties of the small molecule included in the CSM-Lig signatures. Properties were calculated with the Python RDKit library.

Property	Numerical type
Molecular weight	Real
Heavy atoms	Integer
LogP [1]	Real
#Acceptors	Integer
#Donors	Integer
#Heteroatoms	Integer
#Rotatable bonds	Integer
#Rings	Integer
Labute's Approximate Surface Area	Real
Topological Polar Surface Area	Real

Table 2: List of methods used in comparative experiments.

Method/Scoring Function	Reference
RF-Score::Elem-v2	[2]
RF-Score::Elem-v1	[3]
X-Score::HMScore	[4]
DrugScore^{CSD}	[5]
SYBYK::ChemScore	[6]
DS::PLP1	[7]
GOLD::ASP	[8]
SYBYL::G-Score	[9]
DS::LUDI3	[10]
DS::LigScore2	[11]
GlideScore-XP	[12]
DS::PMF	[13]
GOLD::ChemScore	[14]
SYBYL::D-Score	[9]
IMP::RankScore	[15]
DS::Jain	[16]
GOLD::GoldScore	[17]
SYBYL::PMF-Score	[9]
SYBYL::F-Score	[9]

3 CSM-lig Approach

CSM-lig is an efficient machine learning approach for assessing and comparing protein-small molecule affinities from solved structures. The method calculates structural features using the CSM algorithm [18] (called signatures) that together with experimental data are used as evidence to train and test predictive models.

A series of graph-based signatures can be achieved by modelling proteins/small molecule recognition as atomic graphs. The CSM algorithm will then extract distance patterns between its components as described in Algorithm 1.

Algorithm 1 Cutoff Scanning Matrix (CSM) calculation

```
function CSM_lig(LigandSet, AtomClass, D_MIN, D_MAX, D_STEP)
    for all Ligand i ∈ (LigandSet) do
        LigandPocket = extractLigandPocket(Ligand)
        j = 0
        distMatrixInter ← calculateAtomicPairwiseDistInter(LigandPocket)
        for dist ← D_MIN; to D_MAX; step D_STEP do
            for all Class ∈ (AtomClass) do
                CSM_lig[i][j] ← getFrequency(distMatrixInter, dist, class)
                j++
        distMatrixIntra ← calculateAtomicPairwiseDistIntra(LigandPocket)
        for dist ← D_MIN; to D_MAX; step D_STEP do
            for all Class ∈ (AtomClass) do
                CSM_lig[i][j] ← getFrequency(distMatrixIntra, dist, class)
                j++
        addLigandProperties(CSM_lig[i])
    return CSM_lig
end
```

The Algorithm receives a set of protein-ligand complexes, a set of atom classes and distance cutoffs (minimum, maximum and a cutoff step). The ligand pockets are extracted and a cummulative distribution of atoms within each distance (based on the cutoff parameters) is calculated per atom class. The distances are calculated separately in two components: intra-pocket distances, which aim to model pocket geometry and physicochemical properties and inter-complex distances, which aim to account for protein-ligand interactions. After this process, a set of ligand properties (Table 1 of Supplementary Material) are calculated and appended to the signatures.

References

[1] Scott A Wildman and Gordon M Crippen. Prediction of physicochemical parameters by atomic contributions. *Journal of Chemical Information and Computer Sciences*, 39(5):868–873, 1999.

[2] Pedro J Ballester, Adrian Schreyer, and Tom L Blundell. Does a more precise chemical description of protein–ligand complexes lead to more accurate prediction of binding affinity? *Journal of chemical information and modeling*, 54(3):944–955, 2014.

[3] Pedro J Ballester and John BO Mitchell. A machine learning approach to predicting protein–ligand binding affinity with applications to molecular docking. *Bioinformatics*, 26(9):1169–1175, 2010.

[4] Renxiao Wang, Luhua Lai, and Shaomeng Wang. Further development and validation of empirical scoring functions for structure-based binding affinity prediction. *Journal of computer-aided molecular design*, 16(1):11–26, 2002.

[5] Hans FG Velec, Holger Gohlke, and Gerhard Klebe. Drugscorecsd knowledge-based scoring function derived from small molecule crystal data with superior recognition rate of near-native ligand poses and better affinity prediction. *Journal of medicinal chemistry*, 48(20):6296–6303, 2005.

[6] Matthew D Eldridge, Christopher W Murray, Timothy R Auton, Gaia V Paolini, and Roger P Mee. Empirical scoring functions: I. the development of a fast empirical scoring function to estimate the binding affinity of ligands in receptor complexes. *Journal of computer-aided molecular design*, 11(5):425–445, 1997.

[7] Abby L Parrill and M Rami Reddy. *Rational drug design: novel methodology and practical applications.* Number 719. Amer Chemical Society, 1999.

[8] Wijnand Mooij and Marcel L Verdonk. General and targeted statistical potentials for protein–ligand interactions. *Proteins: Structure, Function, and Bioinformatics*, 61(2):272–287, 2005.

[9] Version 7.2. The Sybyl Software. *Triops Inc, St. Loius*, 2006.

[10] Hans-Joachim Böhm. Prediction of binding constants of protein ligands: a fast method for the prioritization of hits obtained from de novo design or 3d database search programs. *Journal of computer-aided molecular design*, 12(4):309–309, 1998.

[11] André Krammer, Paul D Kirchhoff, X Jiang, CM Venkatachalam, and Marvin Waldman. Ligscore: a novel scoring function for predicting binding affinities. *Journal of Molecular Graphics and Modelling*, 23(5):395–407, 2005.

[12] Richard A Friesner, Robert B Murphy, Matthew P Repasky, Leah L Frye, Jeremy R Greenwood, Thomas A Halgren, Paul C Sanschagrin, and Daniel T Mainz. Extra precision glide: docking and scoring incorporating a model of hydrophobic enclosure for protein-ligand complexes. *Journal of medicinal chemistry*, 49(21):6177–6196, 2006.

[13] Ingo Muegge and Yvonne C Martin. A general and fast scoring function for protein-ligand interactions: a simplified potential approach. *Journal of medicinal chemistry*, 42(5):791–804, 1999.

[14] Carol A Baxter, Christopher W Murray, David E Clark, David R Westhead, and Matthew D Eldridge. Flexible docking using tabu search and an empirical estimate of binding affinity. *Proteins: Structure, Function, and Bioinformatics*, 33(3):367–382, 1998.

[15] Hao Fan, Dina Schneidman-Duhovny, John J Irwin, Guangqiang Dong, Brian K Shoichet, and Andrej Sali. Statistical potential for modeling and ranking of protein–ligand interactions. *Journal of chemical information and modeling*, 51(12):3078–3092, 2011.

[16] Ajay N Jain. Scoring noncovalent protein-ligand interactions: a continuous differentiable function tuned to compute binding affinities. *Journal of computer-aided molecular design*, 10(5):427–440, 1996.

[17] Gareth Jones, Peter Willett, Robert C Glen, Andrew R Leach, and Robin Taylor. Development and validation of a genetic algorithm for flexible docking. *Journal of molecular biology*, 267(3):727–748, 1997.

[18] D. E. V. Pires, D. B. Ascher, and T. L. Blundell. mCSM: predicting the effects of mutations in proteins using graph-based signatures. *Bioinformatics*, 30(3):335–342, 2014.
